# PRODH/POX-Dependent Celecoxib-Induced Apoptosis in MCF-7 Breast Cancer

**DOI:** 10.3390/ph14090874

**Published:** 2021-08-29

**Authors:** Magdalena Misiura, Ilona Ościłowska, Katarzyna Bielawska, Jerzy Pałka, Wojciech Miltyk

**Affiliations:** 1 Department of Analysis and Bioanalysis of Medicines, Medical University of Bialystok, Kilińskiego1, 15-089 Bialystok, Poland; magdalena.misiura@umb.edu.pl (M.M.); katarzyna.bielawska@umb.edu.pl (K.B.); 2 Department of Medicinal Chemistry, Medical University of Bialystok, Kilińskiego 1, 15-089 Bialystok, Poland; ilona.zareba@gmail.com (I.O.); pal@umb.edu.pl (J.P.)

**Keywords:** PRODH/POX, proline, breast cancer, apoptosis

## Abstract

Celecoxib (Cx), an inhibitor of cyclooxygenase 2, induces apoptosis of cancer cells. However, the mechanism of the chemopreventive effect remains not fully understood. We aimed to investigate the role of PRODH/POX that is involved in the regulation of apoptosis induced by celecoxib. MCF-7 breast cancer cell line and the corresponding MCF-7 cell line with silenced PRODH/POX (MCF-7^shPRODH/POX^) were used. The effects of Cx on cell viability, proliferation, and cell cycle were evaluated. The expressions of protein markers for apoptosis (Bax, caspase 9, and PARP) and autophagy (Atg5, Beclin 1, and LC3A/B) were investigated by Western immunoblotting. To analyze the proline metabolism, collagen biosynthesis, prolidase activity, proline concentration, and the expression of proline-related proteins were evaluated. The generation of ATP, ROS, and the ratio of NAD+/NADH and NADP+/NADPH were determined to test the effect of Cx on energetic metabolism in breast cancer cells. It has been found that Cx attenuated MCF-7 cell proliferation via arresting the cell cycle. Cx induced apoptosis in MCF-7 breast cancer cells, while in MCF-7^shPRODH/POX^, autophagy occurred more predominantly. In MCF-7 breast cancer cells, Cx affected proline metabolism through upregulation of proline biosynthesis, PRODH/POX and PYCRs expressions, PEPD activity, and downregulation of collagen biosynthesis. In MCF-7^shPRODH/POX^ clones, these processes, as well as energetic metabolism, were remarkably suppressed. The data for the first time suggest that celecoxib induces apoptosis through upregulation of PRODH/POX in MCF-7 breast cancer cells.

## 1. Introduction

One of the chemopreventive approaches to cancer development is the administration of non-steroidal anti-inflammatory drugs (NSAIDs), such as celecoxib (Cx), which are inhibitors of cyclooxygenase 2 (COX-2). Many authors have shown that the administration of NSAIDs inhibits the development of various types of cancers [1]. Apart from analgesic and antipyretic properties, the drug also reduces the number of polyps in patients with familial adenomatous polyposis. Moreover, it was found that celecoxib induces apoptosis of cancer cells via activating PPARγ, inhibits the expression of the EGFR-downstream signaling pathway, as well as HIF-1α activity and HIF-1α-dependent molecules, e.g., VEGF [2,3,4]. The mechanism of the chemopreventive effect of celecoxib is not fully understood. Our previous research shows that this drug modulates proline metabolism in oral squamous cells leading to induction of apoptosis [5]. Breast cancer is a leading cause of death in women based on GLOBOCAN reports [6]. Therefore, the model of estrogen-dependent MCF-7 cell line served as a model for breast cancer. 

Proline oxidase/proline dehydrogenase (PRODH/POX), EC:1.5.5.2, is a mitochondrial enzyme that catalyzes the conversion of proline (Pro) to pyrrolidine-5-carboxylic acid (P5C) [7]. The reverse reaction of P5C conversion into proline by pyrroline-5-carboxylate reductase (PYCR) using NADPH/NADH is linked to the pentose phosphate pathway and glucose metabolism [8]. The oxidation of proline by PRODH/POX is accompanied by a transfer of electrons to the electron transport chain producing ATP or reactive oxygen species (ROS). It is believed that PRODH/POX-dependent generation of ATP enables cell survival, while that of ROS induces both intrinsic and extrinsic apoptosis [7]. The molecular mechanism of switching the PRODH/POX function between introducing cells into the autophagy or apoptosis needs further investigation. It was reported that cancer cells accumulate proline in higher concentrations than healthy cells providing substrate for PRODH/POX activity [5].

An important source of proline constitutes collagen, the most abundant protein in the extracellular matrix. Collagen degradation products are hydrolyzed to free amino acids in lysosomes, apart from imidodipeptides, e.g., glycyl-proline. Imidodipeptides are degraded to free amino acids (glycine, proline) by cytoplasmic imidodipeptidase, prolidase (PEPD) [EC:3.4.13.9] [9]. The mechanisms utilizing proline are either collagen biosynthesis or proline cycle in mitochondrion [10]. PEPD, besides its enzymatic activity, may regulate transcription factors in tumor cells. It was reported that PEPD overexpression causes an increase in the activity of HIF-1α and VEGF that are involved in angiogenesis [11]. The link between PEPD-PRODH/POX-proline-collagen biosynthesis axis and apoptosis mode may explain the mechanism underlying the chemopreventive activity of celecoxib in MCF-7 breast cancer cells. We aimed to investigate the role of PRODH/POX that are involved in the regulation of apoptosis by celecoxib. 

## 2. Results

### 2.1. Celecoxib Limits MCF-7 Cell Proliferation via Arresting the Cell Cycle

**Figure 1:**
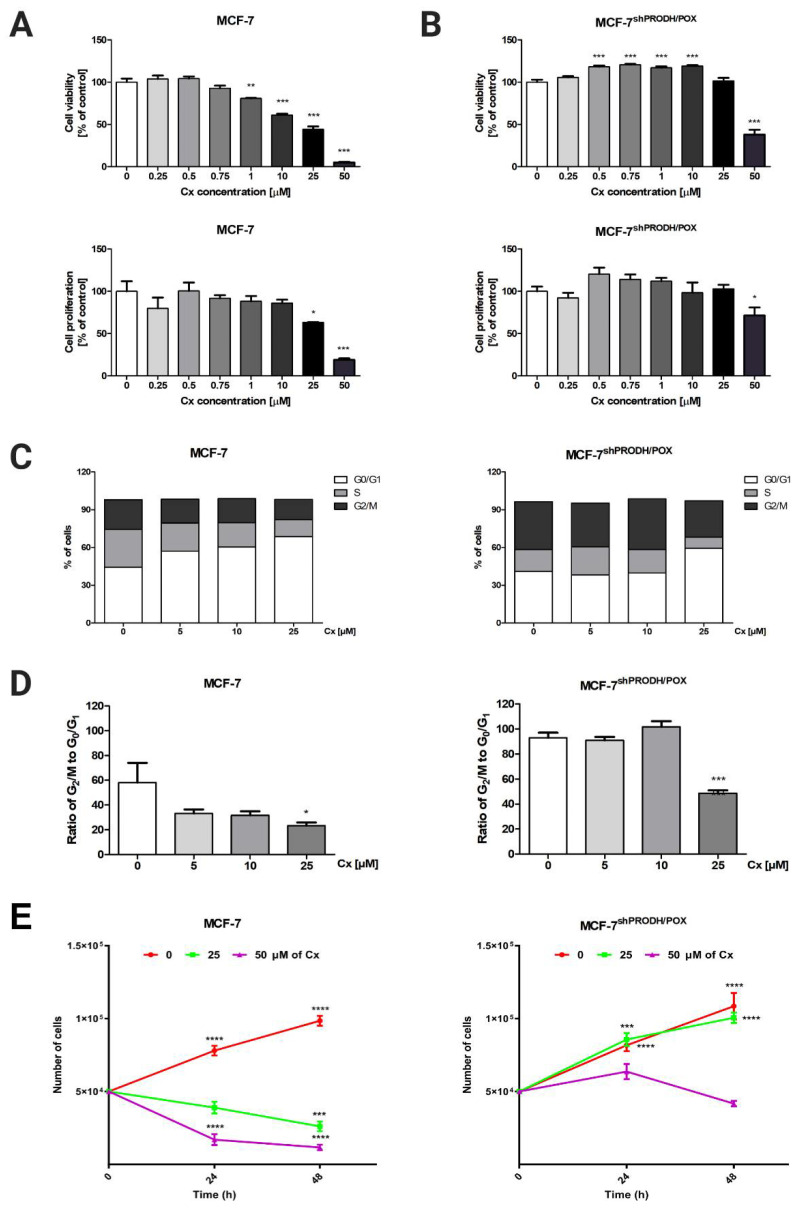
Celecoxib limits MCF-7 cell proliferation via arresting the cell cycle: (**A**) cell viability measured by Cell Titer Blue assay; (**B**) cell proliferation was studied by CyQuant Cell Proliferation Assay and the mean values were presented as a percent of control. Cells were treated with various concentrations of Cx (0, 0.25, 0.5, 1, 5, 10, 25, and 50 µM) for 24 h. * *p* < 0.05, ** *p* < 0.01 and *** *p* < 0.001; (**C**) the percentage of cells in G_0_/G_1_, S and G_2_/M phases of the cell cycle of MCF-7 and MCF-7^shPRODH/POX^ cells; (**D**) the ratio of cell percentage in G_2_/M to G_0_/G_1_ phase. * *p* < 0.05, ** *p* < 0.01 and *** *p* < 0.001; (**E**) The growth curve of cell treated with various concentrations of Cx (0, 25, and 50 µM) for 24 and 48 h; * *p* < 0.05, ** *p* < 0.01, *** *p* < 0.001, and **** *p* < 0.0001.

To investigate the cytotoxic effect of celecoxib (Cx) in breast cancer cells, MCF-7 breast cancer cell line and the corresponding MCF-7 cell line with silenced PRODH/POX (MCF-7^shPRODH/POX^) cells were treated with Cx at increasing concentrations (0.25–50 µM) for 24, 48, and 72 h. Measuring mitochondrial activity and DNA biosynthesis in those cells, we observed that Cx showed dose- and time-dependent antiproliferative activity in both MCF-7 and MCF-7^shPRODH/POX^ cells (Figure 1A,B, Figures S1 and S2). However, these effects were slightly pronounced in MCF-7^shPRODH/POX^ cells after incubation with Cx. Based on cell viability and proliferation tests, we further investigated the biological effects of Cx at concentrations of 5, 10, and 25 µM. The mechanisms underlying the Cx-mediated significant limitation of cell proliferation were analyzed by the analysis of cell cycle progression, applying image cytometry in MCF-7 and MCF-7^shPRODH/POX^ cells. In MCF-7 cells, the cell percentage of G_0_/G_1_ phase (68.8 ± 1.7% vs. 44.3 ± 5.3%) in the Cx-treated group (25 µM) was significantly increased, while the cell percentage of S (13.5 ± 1.3% vs. 30.0 ± 7.3%) and G_2_/M (15 ± 2.6% vs. 23.6 ± 6.2%) phase was decreased, as compared to non-treated cells. In MCF-7^shPRODH/POX^ cells, the cell percentage of G_0_/G_1_ phase (59.3 ± 0.7% vs. 40.9 ± 0.7%) in the Cx-treated group (25 µM) was significantly increased, while the cell percentage of S (8.9 ± 1.3% vs. 17.5 ± 2.2%) and G_2_/M (28.9 ± 1.7% vs. 38.0 ± 1.8%) phase was decreased, as compared with non-treated cells (Figure 1C and Figure S3). To indicate the ratio of dividing cells to non-dividing cells, we plotted the cell percentage of the G2/M phase to this in G0/G1 phase. Figure 1D shows that Cx-treated groups (25 µM) demonstrated significantly halted cell proliferation. These results were confirmed by cell growth curves (Figure 1E).

### 2.2. Celecoxib-Induced Apoptosis in MCF-7 Breast Cancer Cells

**Figure 2:**
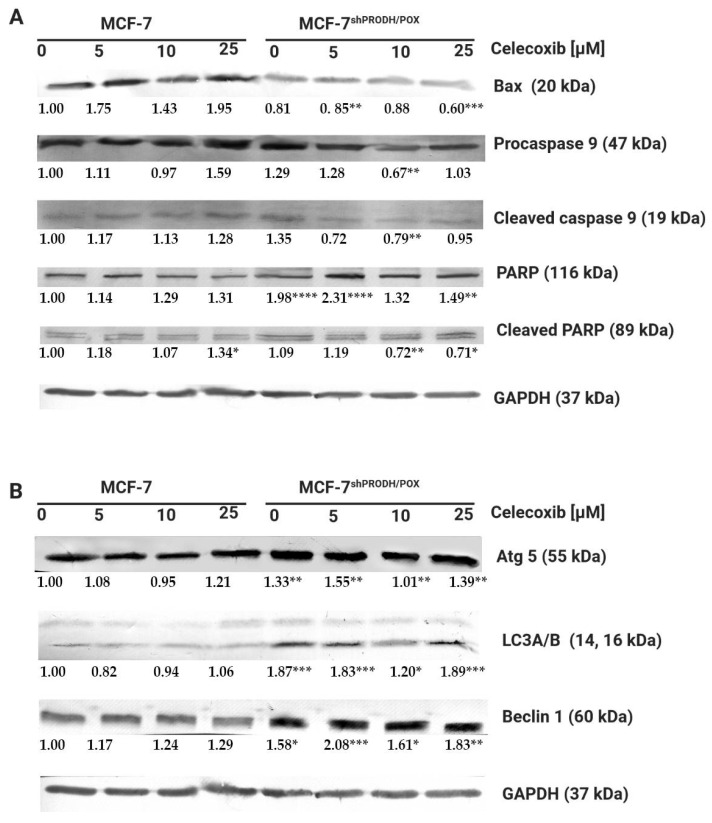
Celecoxib induces apoptosis in MCF-7, while in MCF-7^shPRODH/POX^ cells, autophagy occurs: (**A**) Western blot analysis of protein markers for apoptosis; (**B**) Western blot analysis of protein markers for autophagy. Representative blot images were shown (the mean value of densitometric analysis of protein bands presented below each blot; * *p* < 0.05, ** *p* < 0.01 and *** *p* < 0.001, **** *p* < 0.0001). Supplementary Materials contain statistical analysis of the evaluated proteins.

Firstly, we assessed whether celecoxib affected apoptosis in MCF-7 and MCF-7^shPRODH/POX^ cells. We determined the biological effects of Cx on the expression of the selected proteins involved in the apoptotic pathway using Western immunoblotting analysis. Apoptosis-related proteins Bax, caspase 9, and PARP as apoptosis markers were evaluated. Figure 2A shows the changes in levels of the proteins after treatment with Cx for 24 h. The expression level of the Bax protein increased in MCF-7, while in MCF-7^shPRODH/POX^, the cells remained unchanged. The expression of caspase 9 and its cleaved form was significantly increased in MCF-7 cells incubated with 25 µM celecoxib for 24 h. We observed a markedly decreased expression of PARP and increased expression of cleaved PARP in MCF-7 cells. In MCF-7^shPRODH/POX^ cells, the cleavage of PARP was weakly demonstrated. These results indicate a dose-dependent pro-apoptotic effect of celecoxib on MCF-7 cells, while in MCF-7^shPRODH/POX^ cells, this process was limited. 

It is known that PRODH/POX contributes to autophagic phenotype in breast cancer cells [12]. Herein induction of autophagy was evaluated by western immunoblotting to detect Beclin1, microtubule-associated protein light chain-A and -B(LC3A/B), and Atg5 protein levels. In MCF-7^shPRODH/POX^ cells, autophagy markers were expressed more intensely than in MCF-7 cells. However, Cx treatment did not markedly affect the expression of the selected proteins (Figure 2B and Figure S4). 

The results show Cx induces apoptosis in a dose-dependent manner in MCF-7 cells expressing PRODH/POX, suggesting the crucial role of the protein in the induction of apoptosis.

### 2.3. Celecoxib Affects Proline Metabolism in MCF-7 Breast Cancer Cells

**Figure 3:**
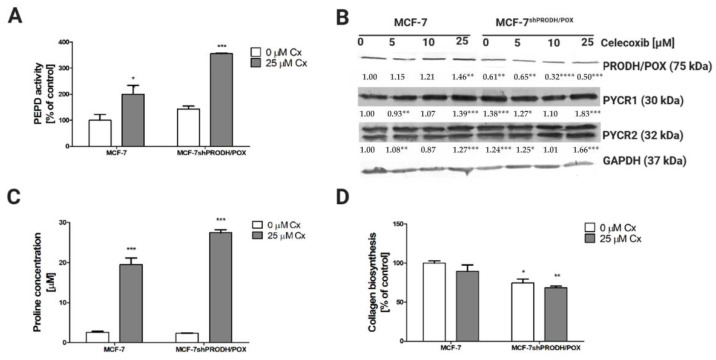
Celecoxibaffects proline metabolism in breast cancer cells: (**A**) prolidase activity after treatment with Cx for 24 h in MCF-7 and MCF-7^shPRODH/POX^ cells; (**B**)Western blot analysis of proteins related to proline cycle; representative blot images were shown (the mean value of densitometric analysis of protein bands presented below each blot; * *p* < 0.05, ** *p* < 0.01, and *** *p* < 0.001, **** *p* < 0.0001). Supplementary Materials contain statistical analysis of the evaluated proteins; (**C**) LC–MS analysis of proline in Cx-treated MCF-7 and MCF-7^shPRODH/POX^ cells after 24 h treatment; (**D**) collagen biosynthesis after treatment with Cx for 24 h. * *p* < 0.05, ** *p* < 0.01, and *** *p* < 0.001.

Since the mechanism underlying the Cx-induced cytotoxic effects remains not fully understood, we proposed a hypothesis that proline metabolism is involved. Proline is a unique amino acid associated with collagen turnover and PEPD activity. The amino acid constitutes 10% of all amino acids in collagen structure and is supplied by PEPD that cleaves C-terminal proline-containing dipeptides [7]. To evaluate whether proline metabolism is related to Cx-induced apoptosis, we assessed prolidase activity, which was significantly increased upon treatment with Cx. Its enzymatic activity was significantly increased in MCF-7 and MCF-7^shPRODH/POX^ cells (Figure 3A). Another mechanism responsible for providing proline is proline reductase (PYCR)-dependent. In MCF-7 cells, we observed an increase in PYCR expression; however, the PYCR1 expression was strongly pronounced compared to that of PYCR2. It is also worth mentioning that, in MCF-7^shPRODH/POX^ clones, the level of PYCRs was significantly higher than in MCF-7 cells (Figure 3B and Figure S5). An increase in the level of expression of these enzymes (PYCR1 and PYCR2) may result in the enhanced conversion of P5C to proline. In MCF-7 cells, we found that Cx at 25 µM stimulated PRODH/POX expression by approximately 40%, while at the same concentration of Cx, its expression in MCF-7^shPRODH/POX^ cell line was decreased. Increased PEPD activity likely led to an increased proline concentration (Figure 3C). Proline serves as a building block for collagen biosynthesis; however, we observed a decrease in this process in Cx-treated cells (Figure 3D). 

### 2.4. Celecoxib Effects on ATP, ROS, Ratios of NAD+/NADH, and NADP+/NADPH in MCF-7 Breast Cancer Cells

**Figure 4:**
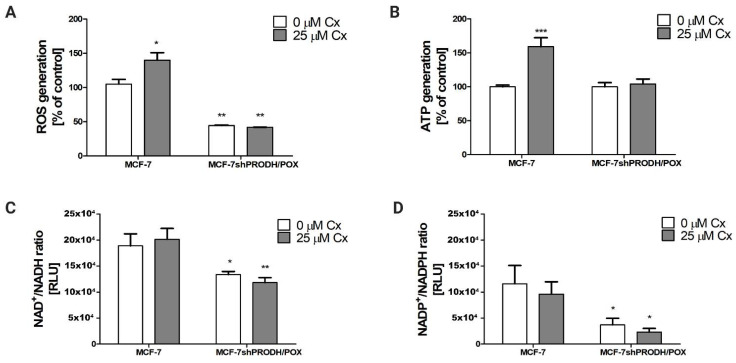
Celecoxib impacts on energetic metabolism in MCF-7 and MCF-7^shPRODH/POX^ cell lines. (**A**) ROS generation; (**B**) ATP generation; (**C**) NAD+/NADH; (**D**) NADP+/NADPH ratios after 24 h treatment of the cells with Cx. * *p* < 0.05, ** *p* < 0.01 and *** *p* < 0.001.

It is known that PRODH/POX leads to ATP and ROS generation, supporting autophagy and apoptosis, respectively [13]. Energetic metabolism includes pentose phosphate cycle components such as NAD^+^, NADH, NADP^+^, and NADPH, which serve for dinucleotide synthesis and cell proliferation. In MCF-7 cells, we detected a significant increase in ATP and ROS generation after 24 h treatment with Cx (Figure 4A,B). In cells carrying silenced PRODH/POX, the production of these molecules was markedly suppressed. In Cx-treated MCF-7 cells, the ratios NAD^+^/NADH and NADP^+^/NADPH remained unchanged compared to the control (Figure 4C,D). However, we discovered that these parameters decreased significantly in MCF-7^shPRODH/POX^ cells. The data suggest that induction of PRODH/POX by Cx correlates with the increase in the indicated parameters of energetic metabolism in breast cancer cells.

**Figure 5:**
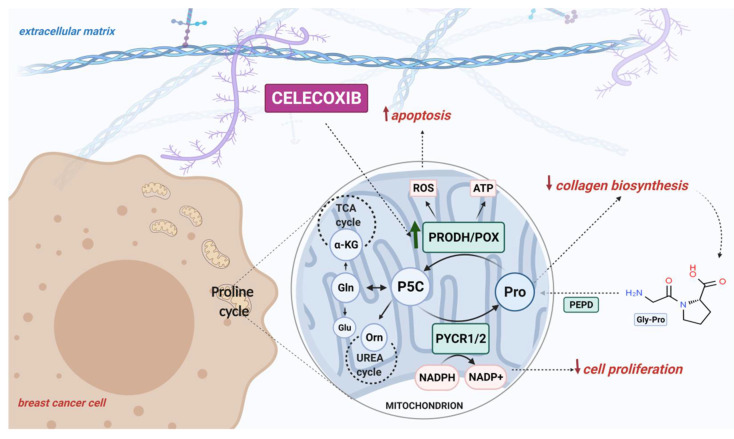
Schematic overview of the PRODH/POX-dependent celecoxib-induced apoptosis in MCF-7 breast cancer cells. Created with BioRender.com.

Our finding of Cx-mediated regulation of PRODH/POX-dependent apoptosis in an experimental model of breast cancer cells is summarized in Figure 5. 

## 3. Discussion

Celecoxib is a selective COX-2 inhibitor, and it is known that Cx exhibits anticancer effects in models in vitro and in vivo, including colon, lung, and prostate cancer; however, its anticancer activity involves COX-dependent and -independent mechanisms [13]. To the best of our knowledge, this is the first study presenting celecoxib-mediated apoptosis through the PRODH/POX pathway in breast cancer cells. In this study, we provided a comprehensive insight into the cellular metabolism of celecoxib-treated breast cancer cells, since the mechanism explaining the mode of Cx activity remains not fully understood. We propose a hypothesis that PRODH/POX serves as a molecular effector for celecoxib-induced apoptosis in MCF-7 breast cancer cells. 

Our study demonstrates that celecoxib exhibited antiproliferative activity in MCF-7 breast cancer cells via arresting cell cycle in the G_0_/G_1_ phase. This is in line with the study of Dai et al. [14]. However, our data contribute to expanding an understanding of Cx-mediated cell arrest through PRODH/POX induction. Having demonstrated that Cx blocks cell cycle progression in MCF-7 breast cancer cells, we further investigated whether PRODH/POX affected the cell cycle. In MCF-7^shPRODH/POX^ cells with silenced PRODH/POX, cell cycle arrest in the G_0_/G_1_ phase occurred less effectively, suggesting that PRODH/POX is a crucial player in this process. Celecoxib arrested cell cycle in the G_0_/G_1_ phase in MCF-7 cells, while the effect in MCF-7^shPRODH/POX^ was much less noticeable. These results provide evidence of a pro-apoptotic function of PRODH/POX [10,12], suggesting that this pathway could contribute to the development of anticancer therapies as well as additional explanation of the mechanism of celecoxib chemopreventive activity. Enhanced cell cycle blockage drives apoptosis. Herein, induction of apoptosis occurred in Cx-treated MCF-7 cells, where the expression of Bax, caspase 9, and PARP, as markers of apoptosis, was more pronounced. In MCF-7^shPRODH/POX^, autophagy predominantly occurs to sustain cell survival. Currently, the role of autophagy is controversial in cancer progression, since its regulation in cancer cells is complex. Autophagy can lead to tumor cell survival under multiple stresses, while inhibition of autophagy can drive tumorigenesis [15]. 

Our results indicated that Cx stimulated the expression of PRODH/POX in MCF-7 cells. Similar results were presented in in vitro study [5], showing that Cx upregulates PRODH/POX in oral squamous cell carcinoma. The authors also demonstrated that the expression of this enzyme was significantly diminished in oral squamous cell carcinoma tissue specimens. A downregulation of PRODH/POX also occurs in kidney and digestive tract cancers, contributing to cancer development and progression [16]. However, the studies indicate that the pattern of PRODH/POX expression differs in various cancers. In prostate cancer, lung, and pancreatic ductal adenocarcinoma, the expression of PRODH/POX was upregulated [17,18,19], but was downregulated in the human digestive tract and kidney tumors [18]. Liu et al. found that overexpression of PRODH promotes epithelial-to-mesenchymal transition (EMT) in non-small cell lung carcinoma, and its inhibition causes a limitation of cell proliferation and EMT [19]. Taken together, PRODH/POX has distinct functions depending on the tumor microenvironment.

In the study, we presented Cx upregulated PRODH/POX, prolidase activity, and proline availability, while collagen biosynthesis was downregulated. Proline is mainly involved in collagen synthesis; however, recent publications indicate its role as a key regulator of various biochemical processes in cellular metabolism. Proline may originate from collagen catabolism or reduction in pyrroline-5-carboxylic acid (P5C). Apart from its role in protein synthesis, proline can act as a signaling molecule, but can also maintain redox balance and serve as an energy source [20]. Liu et al. demonstrated that intracellular proline may be related to increased activity of c-MYC, an oncogenic transcription factor. Its enhanced activity leads to an increase in proline and glutamine biosynthesis [21]. 

The role of proline in redox balance in cancer cell metabolism is reflected by protecting cancer cells from oxidative stress. The mechanism underlying an increase in the antioxidant potential of proline may be explained in two ways: (1) the conversion of proline to glutamate, an intermediate of glutathione, could be accelerated; or (2) proline activates antioxidant enzymes, reducing the generation of reactive oxygen species (ROS). PRODH/POX provides electrons into the electron transport chain to produce ROS or ATP. It has been discovered that PRODH/POX is a tumor suppressor and, by ROS signaling, initiates apoptosis, limits tumor cell growth, and arrests the cell cycle progression [22]. It has been found that antioxidants reduce PRODH/POX-dependent apoptosis in cellular models. Phang et al. [23] tested the influence of N-acetylcysteine (NAC) or enzymes-superoxide dismutase (SOD) and catalase (CAT) on inhibition of PRODH/POX-induced apoptosis. The addition of NAC to the cell culture medium significantly reduced PRODH/POX-induced apoptosis. The induction of MnSOD expression reduced the release of cytochrome c to the cytoplasm and inhibited PRODH/POX-induced apoptosis. Based on the results, the authors stated that the generation of superoxide radicals plays a crucial role in PRODH/POX-induced apoptosis, and the process is partially blocked by SOD or NAC. Moreover, Zamaraeva et al. [24] stated that elevation of the cytosolic ATP level is an absolute requirement for the events in apoptotic cell death. Inhibition of ATP synthesis by oligomycin (suppressor of mitochondrial respiration) halted the initiation step of apoptosis. Imamura et al. showed that the cytosolic ATP level starts to decrease immediately after the activation of caspase-3, and this process is completed typically within 2 h. The ATP decrease was facilitated by caspase-dependent cleavage of the plasma membrane channel pannexin-1 [25]. We found that MCF-7^shPRODH/POX^ cells generated less ATP compared to MCF-7 cells; therefore, we presume that it is caused by lesser requirements for energy since apoptosis does not occur. Programmed cell death involves many ATP-dependent stages, such as enzymatic hydrolysis of macromolecules, caspase activation, chromatin condensation, and apoptotic body formation. In MCF-7^shPRODH/POX^, the proapoptotic effect of Cx was not as potent as in MCF-7 cells; thus, the difference in ATP level in clones was marginal. In addition, proline degradation is not an efficient source for generating ATP; however, its small contribution could be significant in the metabolic adaptation of cancer cells. Proline can be converted to α-ketoglutarate (αKG), which may be used as a supplier to the tricarboxylic acid (TCA) cycle and by oxidative phosphorylation for ATP production [26,27]. Block or deficiency in proline conversion by silenced PRODH/POX expression can disturb bioenergy status, leading to a decrease in ATP level. The results fit with the theory proposed by Elia et al. [28], suggesting that PRODH/POX activity contributes to ATP generation in the 3D culture of breast cancer cells. Further investigations demonstrated that intracellular proline supported the maintaining of ATP at an adequate level. Other players in PRODH/POX-mediated apoptosis induced by Cx are NAD+/NADH and NADP+/NADPH. We found that, in MCF-7^shPRODH/POX^ clones, the generation of NAD+/NADH and NADP+/NADPH was decreased in comparison to MCF-7 cells carrying PRODH/POX. NADP+ and NADPH are necessary when P5C is reduced to proline [29]. The reduction in NAD+/NADH and NADP+/NADPH levels reduces the availability of nucleotides for DNA biosynthesis and, as result, cell proliferation [30]. 

Taken together, stimulation of PRODH/POX by celecoxib contributes to inhibition of breast cancer cell growth through activation of PRODH/POX-dependent apoptotic pathways. Our findings are important not only in proving a celecoxib-based strategy to inhibit cancer growth but also in supporting the concept of a PRODH/POX-dependent mechanism of celecoxib anticancer activity.

## 4. Materials and Methods

### 4.1. Cell Culture and Treatment

Breast cancer cell line MCF-7 was purchased from ATCC (HTB-22, ATCC, Manassas, VA, USA). MCF-7^shPRODH/POX^ cell clones carried shRNA-based *PRODH/POX* knock-down enzymes and were selected according to the previous paper [12]. Cells were cultured in phenol red-containing DMEM cell culture medium (PanBiotech, Aidenbach, Germany) supplemented with 10% fetal bovine serum (FBS; Gibco, Carlsbad, CA, USA) and 1% Penicillin/Streptomycin (Gibco, Carlsbad, CA, USA) at 37 °C in a humidified atmosphere of 5% CO_2_. The medium was replaced every 3 days until confluency. Cells (6–10th passages) were treated with celecoxib (Acros Organics, Geel, Belgium) at the concentrations of 0.25–50 µM for 24, 48, and 72 h in a FBS-free DMEM medium.

### 4.2. Cell Viability Assay

Cell viability of MCF-7 and MCF-7^shPRODH/POX^ cells was measured using Cell Titer Blue assay accordingly to the manufacturer’s protocol (Promega, Madison, WI, USA). Cells were submitted to Cx treatment at concentrations of 0.25–50 µM for 24, 48, and 72 h. Briefly, cells were incubated with a resazurin-containing solution at 37 °C for 2 h. Absorbance was read on TECAN Infinite^®^ M200 PRO (Tecan Group Ltd., Männedorf, Switzerland) at 570 nm and 600 nm as a reference wavelength. The results were presented as a percent of the control value.

### 4.3. Cell Proliferation Assay

The proliferation of MCF-7 and MCF-7^shPRODH/POX^ cells was evaluated using commercially available CyQUANT^®^ Cell Proliferation Assay (Thermo Fisher Scientific, Waltham, MA, USA) according to the manufacturer’s procedure. Cells were treated with Cx at concentrations of 0.25–50 µM for 24, 48, and 72 h. The read was performed on TECAN Infinite^®^ M200 PRO (Tecan Group Ltd., Männedorf, Switzerland) at 480 and 520 nm as excitation and emission wavelengths, respectively. The results were presented as a percent of the control value.

### 4.4. Growth Curve Assay

The number of cells was determined using Nucleo Counter NC-3000 (ChemoMetec, Copenhagen, Denmark). Confluent cells were treated with Cx at concentrations of 25 and 50 µM for 24 and 48 h. The medium was discarded, and the cells were rinsed three times with phosphate-buffered saline (PBS). Then, the cells were harvested, washed, and stained with Solution 13 (ChemoMetec), containing acridine orange (AO) and DAPI, and analyzed using NC-3000 cell counter. The results were presented as a number of the cells.

### 4.5. Cell Cycle Analysis

Analysis of the cell cycle of MCF-7 and MCF-7^shPRODH/POX^ cells was performed as follows. Non-treated and Cx-treated (5–25 µM, 24 h) cells were trypsinized and centrifuged (5 min, 500× *g*) followed by washing twice with PBS. The suspended pellet (500 µL PBS) was fixed in 70% ethanol (4.5 mL) and stored (4 °C) until the day of analysis. After centrifugation (5 min, 500× *g*), ethanol-fixed cells were mixed with Solution 3 (ChemoMetec, Allerod, Denmark), incubated (37 °C, 5 min), and analyzed with an image cytometer (NC-3000, ChemoMetec, Allerod, Denmark).

### 4.6. ATP Production

The production of ATP was measured using commercially available ATP Bioluminescence Assay Kit HS II (Roche, Switzerland). Briefly, Cx-treated cells (24 h) were trypsinized and centrifuged (5 min, 500× g). The pellet was suspended in 0.5 mL dilution buffer and mixed with 0.5 mL cell lysis reagent. Before luminescence analysis, the plate was incubated for 5 min at room temperature (RT) in the dark. The luminescence was read on TECAN Infinite^®^ M200 PRO (Männedorf, Switzerland). The results were presented as a percent of the control value.

### 4.7. Analysis of NAD^+^/NADH and NADP^+^/NADPH

The ratios of NAD/NADH and NADP/NADPH were evaluated using NAD/NADH-Glo™ Assay and NADP/NADPH-Glo™ Assay (Promega, Madison, WI, USA), respectively. The assays were performed according to the manufacturer’s instructions. An equal volume of medium from non-treated and Cx-treated cells for 1 h were mixed with an equal volume of NAD/NADH-Glo™ Detection Reagent (contains reductase, reductase substrate, NAD cycling enzyme, and NAD cycling substrate) or NADP/NADPH-Glo™ Detection Reagent (contains reductase, reductase substrate, NADP cycling enzyme, and NADP cycling substrate). The mixtures were incubated in RT for 1 h. The luminescence was read on a TECAN Infinite^®^ M200 PRO (Tecan Group Ltd., Männedorf, Switzerland).

### 4.8. ROS Production

Generation of ROS was measured by ROS Detection Cell-Based Assay Kit (DCFDA) (Cayman, Ann Arbor, MI, USA). The procedure was performed according to the manufacturer’s protocol. After treatment with Cx, cells were washed with buffer provided by the manufacturer, then mixed with 2′,7′-Dichlorofluorescin diacetate (10 mM) and incubated for 90 min at 37 °C. Fluorescence was read on TECAN Infinite^®^ M200 PRO (Tecan Group Ltd., Männedorf, Switzerland) at 485 and 525 nm as excitation and emission wavelengths, respectively.

### 4.9. Cell Lysate Preparation

Cells were cultured in FBS-free DMEM with Cx (5–25 µM) for 24 h. The procedure for harvesting the cells was performed as previously described [31]. The supernatant was aliquoted and stored at −80 °C. Protein concentration was measured using the Pierce BCA assay kit (Thermo Fisher Scientific, Waltham, MA, USA).

### 4.10. Western Immunoblotting

Western blot analysis was carried out as described by Misiura et al. [31]. The membranes were incubated with primary antibodies diluted 1000 times in 5% bovine serum albumin (Sigma Aldrich, Saint Louis, MO, USA) in TBS-T (20 mM Tris, 150 mM NaCl, 0.1% Tween-20, pH 7.6). Anti-Bax, anti-Caspase 9, anti-PARP, anti-GAPDH, anti-PYCR1, anti-Atg5, anti-LC3A/B I/II, and anti-Beclin 1 were purchased from Cell Signaling Technology, Danvers, MA, USA; anti-PYCR2 and anti-PRODH/POX from St John’s Laboratory, London, UK), followed by incubation with alkaline phosphatase-linked goat anti-rabbit or anti-mouse antibodies (dilution: 1:10,000 in 5% non-fat dried milk (Santa Cruz Biotechnology, Dallas, TX, USA) in TBS-T; Sigma Aldrich, Saint Louis, MO, USA). The bands’ intensities were semi-quantitatively measured in ImageJ software (https://imagej.nih.gov/ij/, accessed on 15 February 2021). All experiments were run at least in triplicates.

### 4.11. Determination of Prolidase Activity

The activity of prolidase was determined according to the method published by Besio et al. [32]. Absorbance was read at 515 nm on TECAN Infinite^®^ M200 PRO (Tecan Group Ltd., Männedorf, Switzerland). The results were reported as a percent of the control value.

### 4.12. Evaluation of Collagen Biosynthesis

Collagen biosynthesis was determined by the incorporation of radioactive 5-[^3^H]-proline at a concentration of 5 μCi/mL (Hartmann Analytic, Braunschweig, Germany) into proteins susceptible to bacterial collagenase according to Peterkofsky’s method [33]. Liquid Scintillation Analyzer Tri-Carb 2810 TR (PerkinElmer, Waltham, MA, USA) was applied for a radiometric read. The results were normalized to total protein biosynthesis and were presented as a percent of the control value.

### 4.13. LC–MS-Based Quantitative Analysis

LC–MS analysis of proline was conducted according to the method of Klupczynska et al. [34]. Methanol-extracted proline was subjected to Agilent 1260 Infinity HPLC system coupled to Agilent 6530 Q-TOF mass spectrometry detector (Agilent Technologies, Santa Clara, CA, USA) with electrospray ionization as an ion source in positive ionization mode. L-proline-d3 (Sigma Aldrich, Saint Louis, MO, USA) was used as an internal standard. A HILIC column (Luna HILIC, 2 × 100 mm, 3 µm, Phenomenex, Torrance, CA, USA) was applied to separate the tested mixture. The samples were collected in three biological repeats, injected in duplicates, and randomized before analysis. Total protein concentration was used for normalization. 

### 4.14. Statistical Analysis

All experiments were carried out at least in three replicates and the experiments were repeated at least three times. Data are shown as a mean ± standard error (SEM). For statistical calculations, one-way analysis of variance (ANOVA) with Dunnett’s correction and *t*-test were used. Statistical analysis was performed using GraphPad Prism 5.01 (GraphPad Software, San Diego, CA, USA). Statistically significant differences were marked as * *p* < 0.05, ** *p* < 0.01, *** *p* < 0.001, **** *p* < 0.0001.

## Data Availability

Data is contained within article and supplementary materials.

## Supplementary Materials

The following are available online at https://www.mdpi.com/article/10.3390/ph14090874/s1, Figure S1: MCF-7 and MCF-7shPRODH/POX cell viability and proliferation after 48 h treatment with a wide range of Celecoxib concentrations. * *p* < 0.05, ** *p* < 0.01 and *** *p* < 0.001. Figure S2: MCF-7 and MCF-7shPRODH/POX cell viability and proliferation after 72 h treatment with a wide range of Celecoxib concentrations. * *p* < 0.05, ** *p* < 0.01 and *** *p* < 0.001. Figure S3: Graphical representation of the cell cycle Analysis upon Cx treatment for 24 h. MCF-7 and MCF-7shPRODH/POX cells were treated with 0, 5, 10 and 25 μM of Cx. Figure S4: Western immunoblots with densitometric analysis presented in Figure 2. Figure S5: Representative blots with densitometric analysis presented in Figure 3. Figure S6: Efficacy of shRNA-based PRODH/POX knock-down in MCF-7 cells. PRODH/POX protein expression in non-treated DLD-1, fibroblasts, MCF-7, and MCF-7shPRODH/POX cell lysates analyzed by Western immunoblotting as previously published [1]. DLD-1 cells were used as a positive control and fibroblasts as a negative control for the expression of PRODH/POX. Transfection of the MCF-7cells with different PRODH/POX shRNA constructs (clone 1-3) were done Representative blot is presented and the intensity of POX bands was quantified by densitometry and normalized to GAPDH, values represent the mean (% of control) ± SD of three experiments, * *p* < 0.001.
