# Supplementary material for: PRODH/POX-Dependent Celecoxib-Induced Apoptosis in MCF-7 Breast Cancer
Source: Pharmaceuticals (Basel). 2021 Aug 29;14(9):874. doi: 10.3390/ph14090874 (PMC8471327; doi:10.3390/ph14090874)
Supplement: Supplementary file 1 [file pharmaceuticals-14-00874-s001.zip › pharmaceuticals-1332259-supplementary.pdf]

Supplementary Materials

PRODH/POX-dependent celecoxib-induced apoptosis in MCF-7 breast cancer.

Magdalena Misiura [1†], Ilona Ościłowska [2†], Katarzyna Bielawska [1], Jerzy Pałka [2] and Wojciech Miltyk [1,*]

[1] Department of Analysis and Bioanalysis of Medicines, Medical University of Bialystok, Kilińskiego 1, 15-089 Bialystok, Poland, phone: +48-85-748-5845; e-mail: magdalena.misiura@umb.edu.pl (M.M.); katarzyna.bielawska@umb.edu.pl (K.B.); wojciech.miltyk@umb.edu.pl (W.M.)

[2] Department of Medicinal Chemistry, Medical University of Bialystok, Kilińskiego 1, 15-089 Bialystok, Poland, phone: +48-85-748-5706; e-mail: ilona.zareba@gmail.com (I.O.); pal@umb.edu.pl (J.P.)

* Correspondence: wojciech.miltyk@umb.edu.pl; Tel.: +48-85-748-5845

† These authors contributed equally to this work.

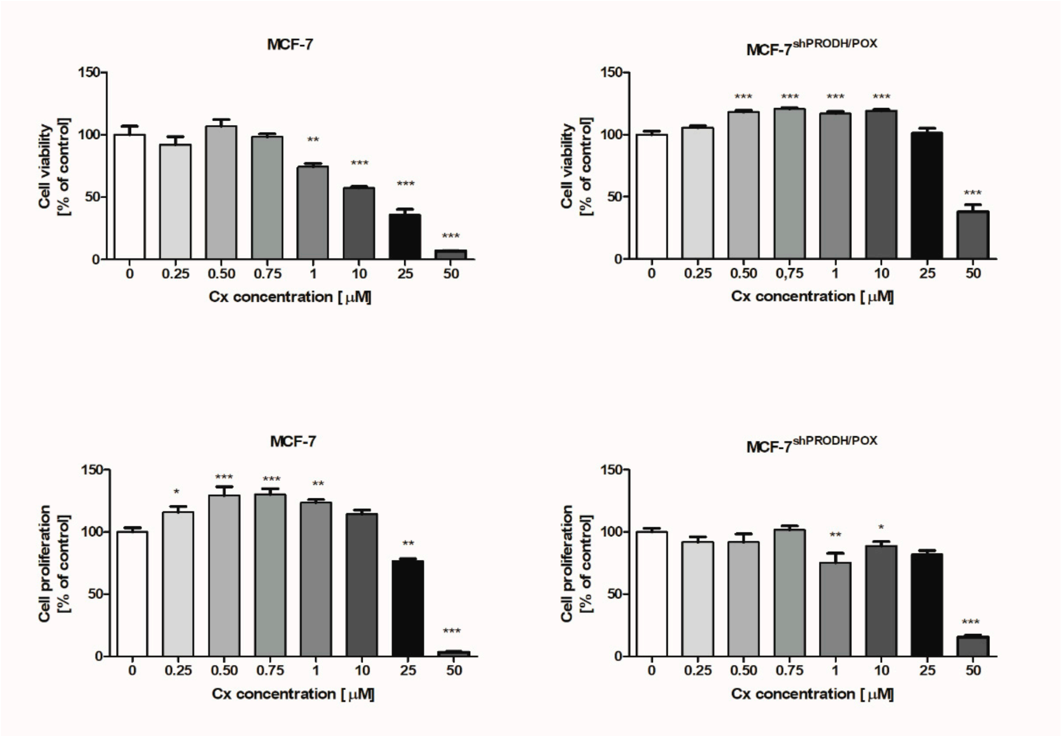

Figure S1. MCF-7 and MCF-7$^{shPRODH/POX}$ cell viability and proliferation after 48 h treatment with a wide range of Celecoxib concentrations. * p<0.05, ** p <0.01 and *** p <0.001.

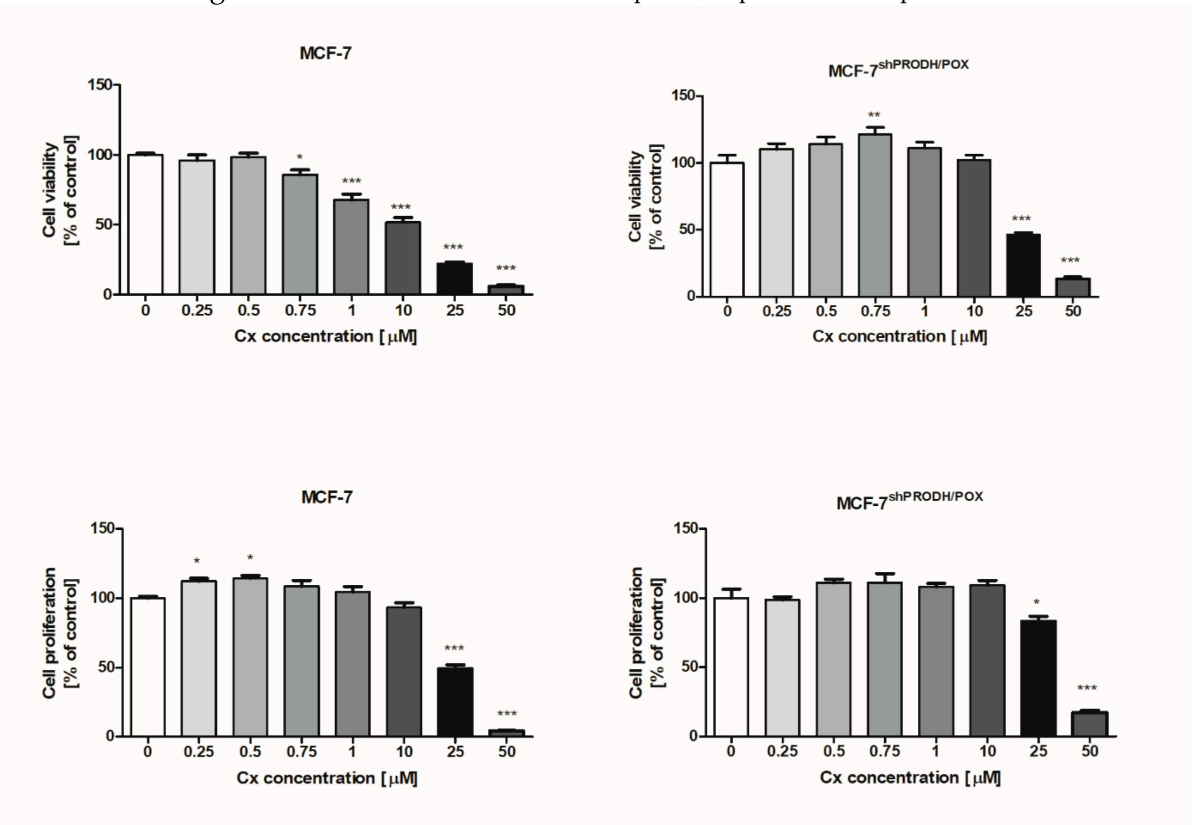

Figure S2. MCF-7 and MCF-7$^{shPRODH/POX}$ cell viability and proliferation after 72 h treatment with a wide range of Celecoxib concentrations. * p<0.05, ** p <0.01 and *** p <0.001.

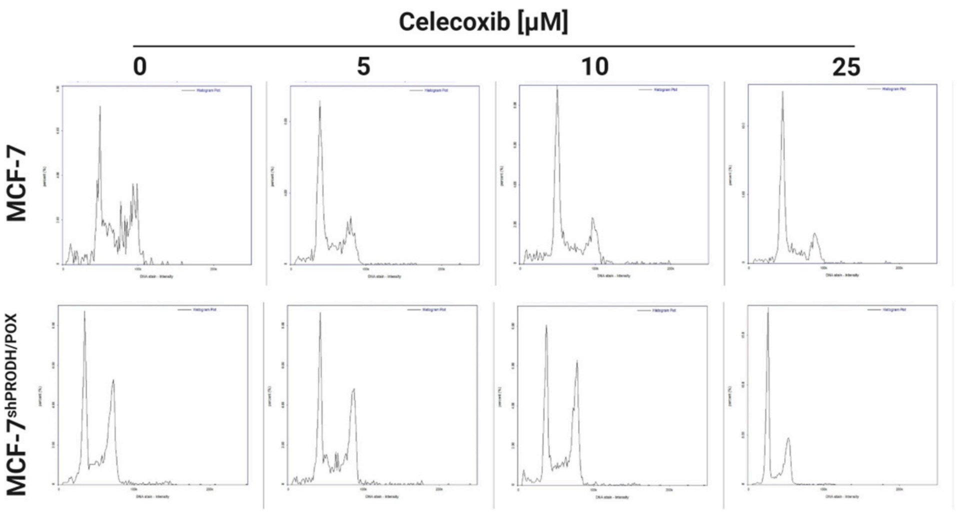

Figure S3. Graphical representation of the cell cycle Analysis upon Cx treatment for 24 h. MCF-7 and MCF-7$^{shPRODH/POX}$ cells were treated with 0, 5, 10 and 25 µM of Cx.

Bax protein expression

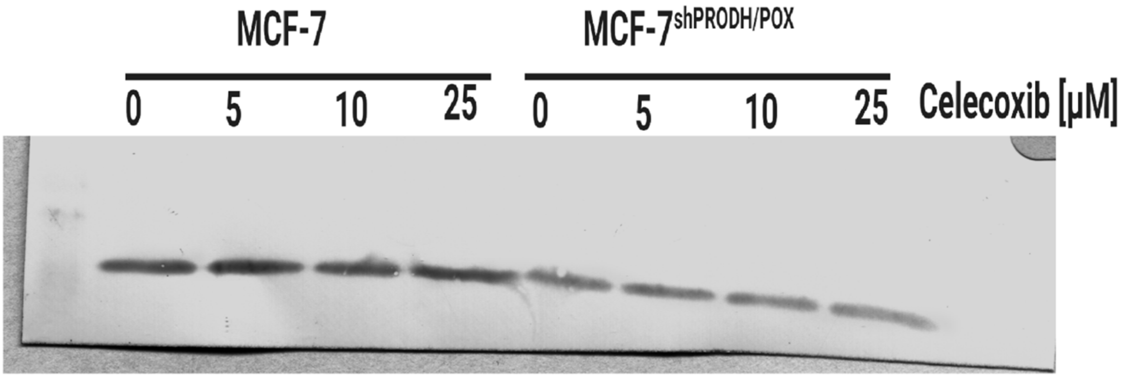

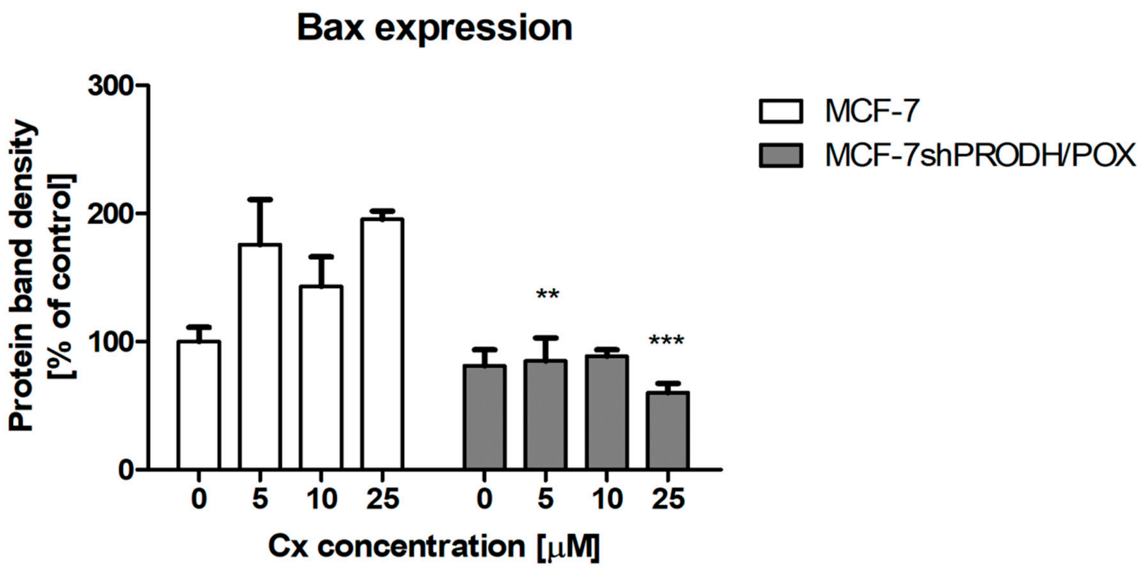

Caspase 9 protein expression

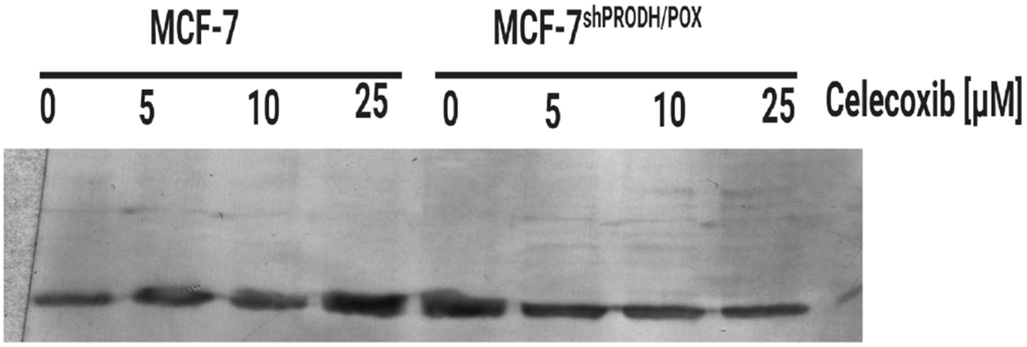

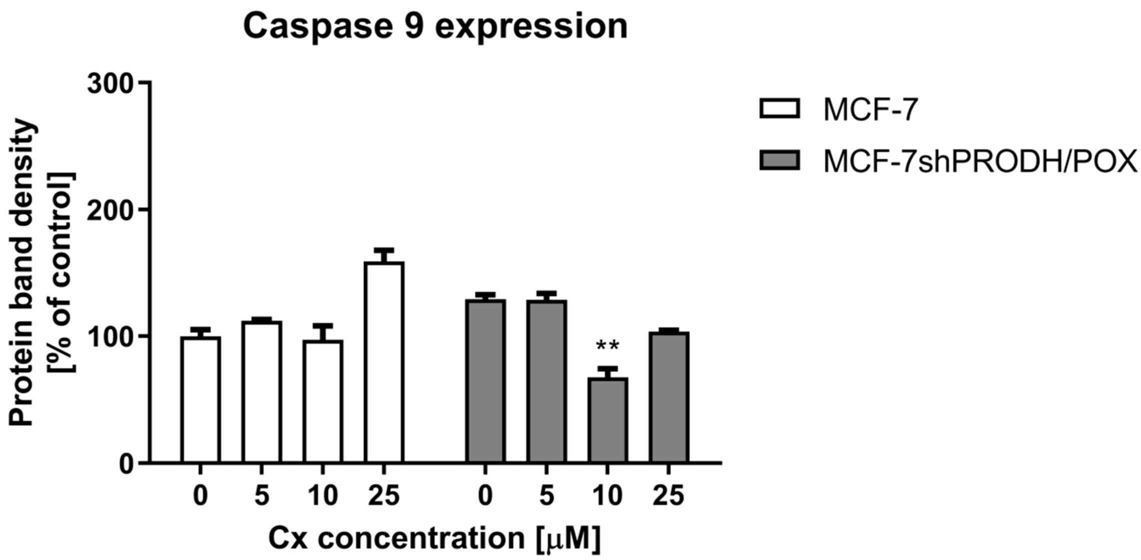

Cleaved Caspase 9 protein expression

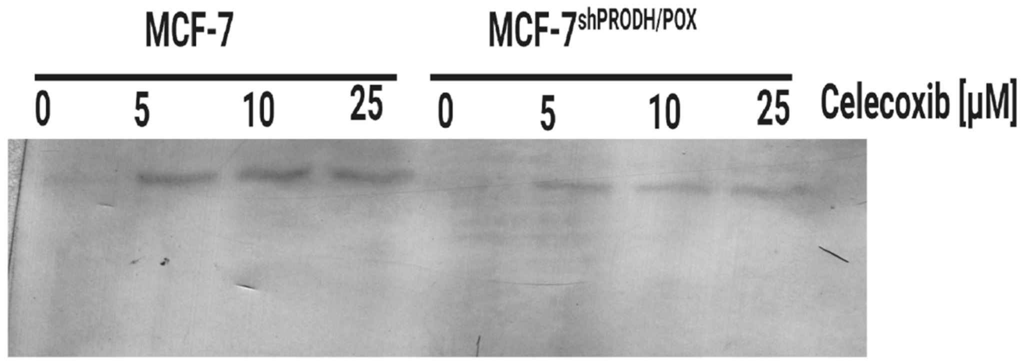

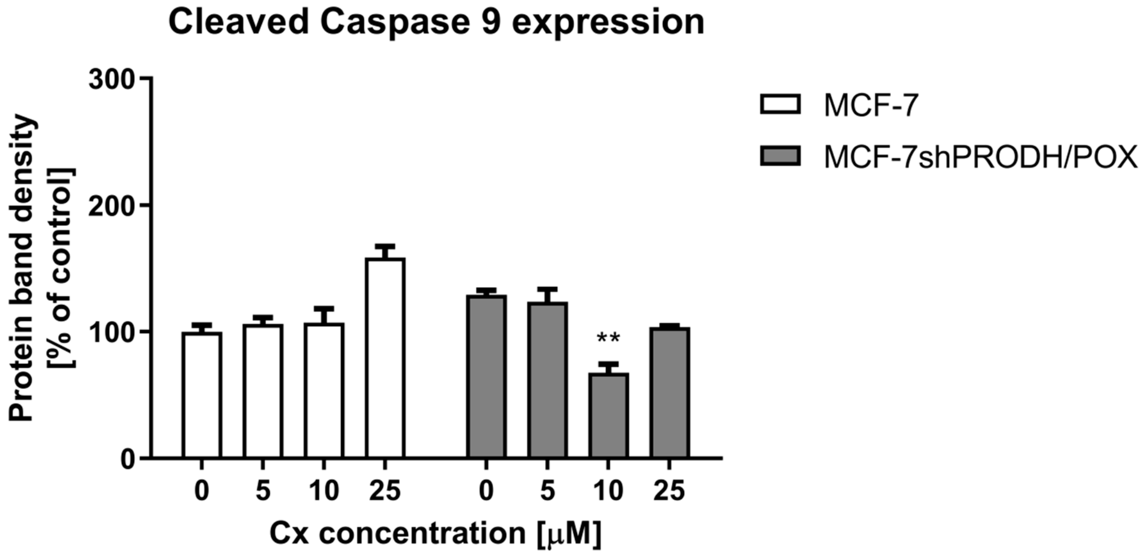

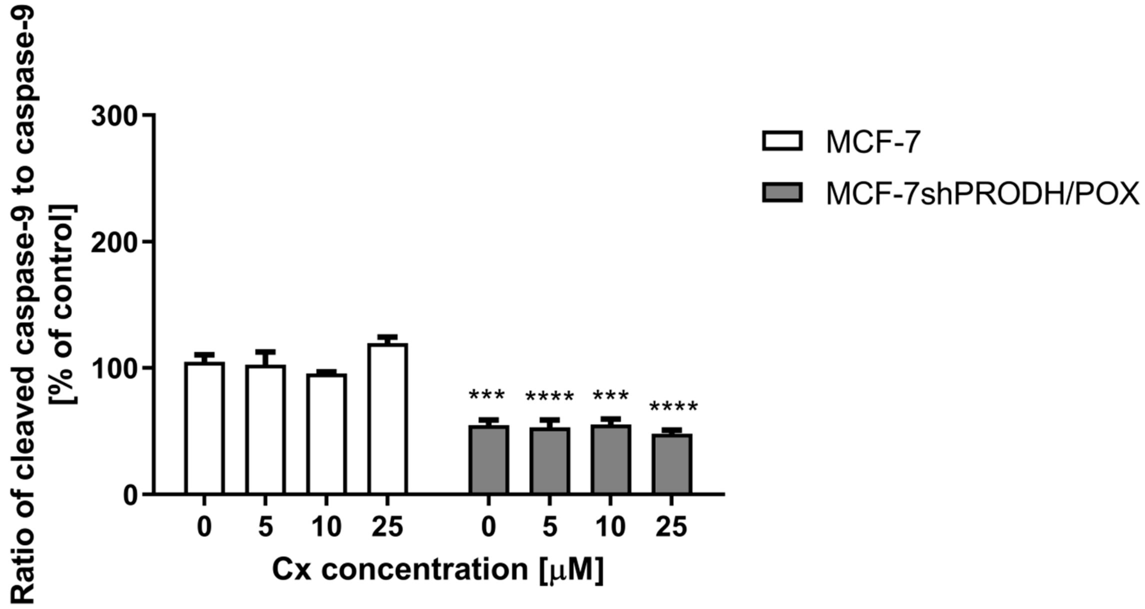

PARP protein expression

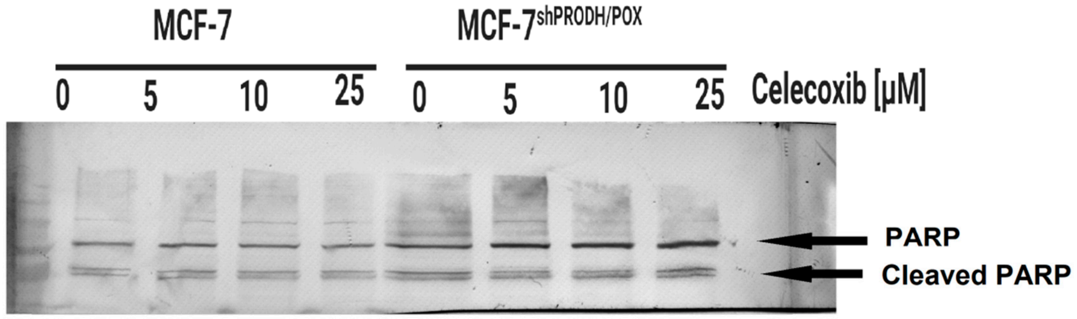

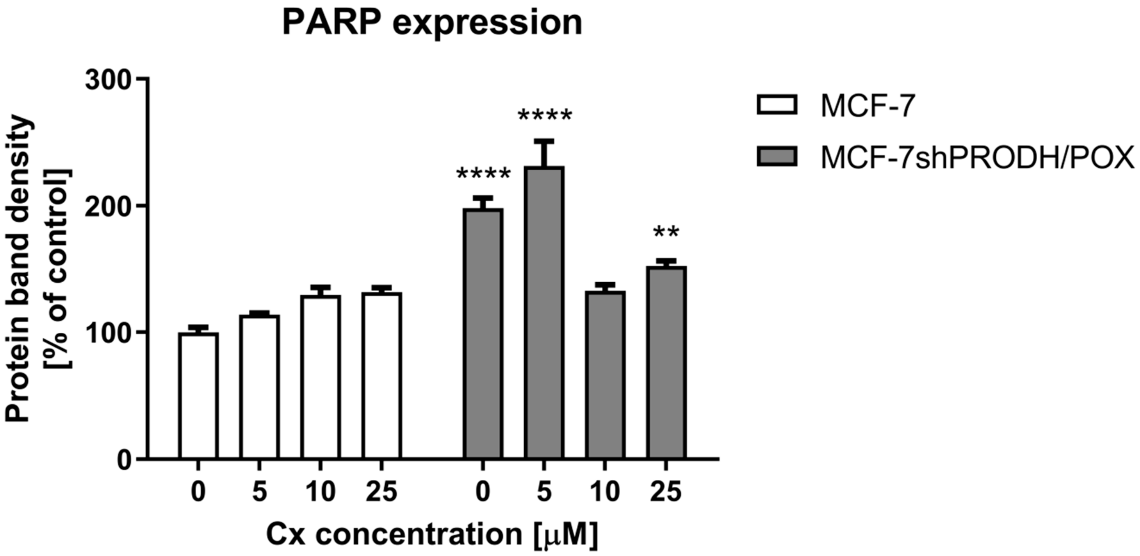

Cleaved PARP protein expression

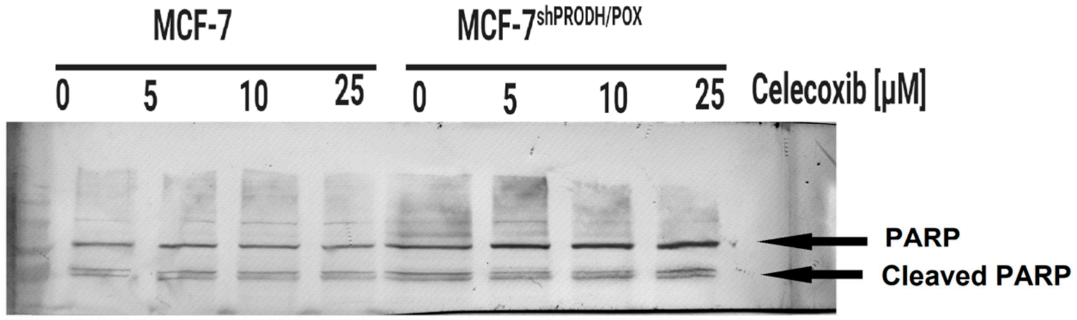

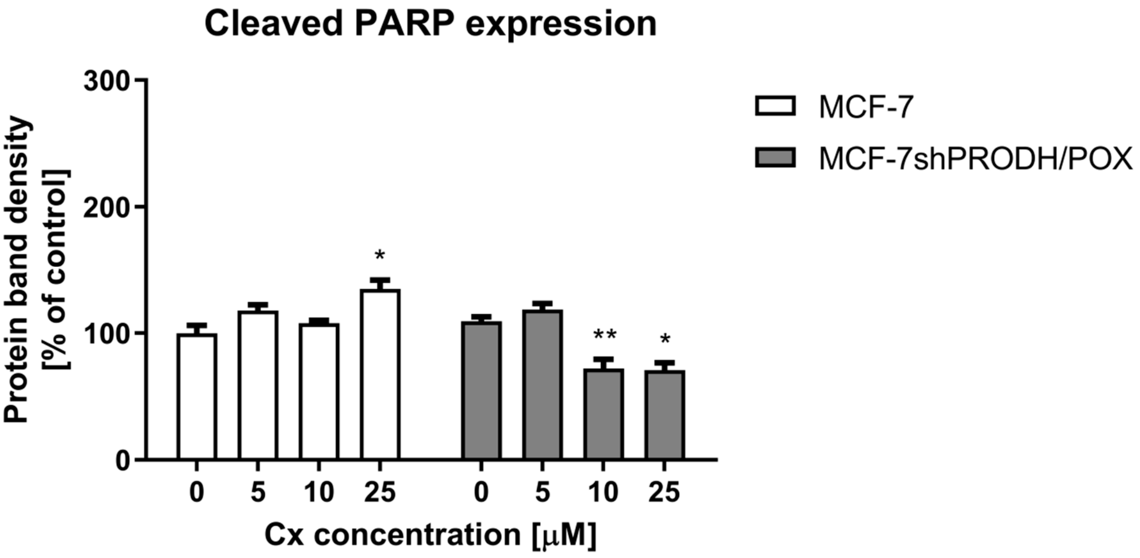

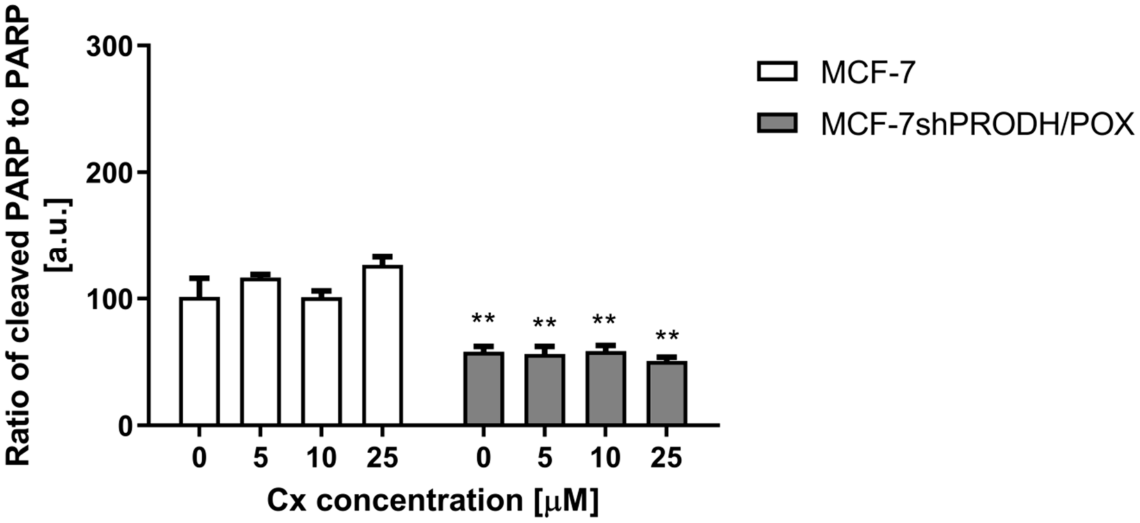

Atg5 protein expression

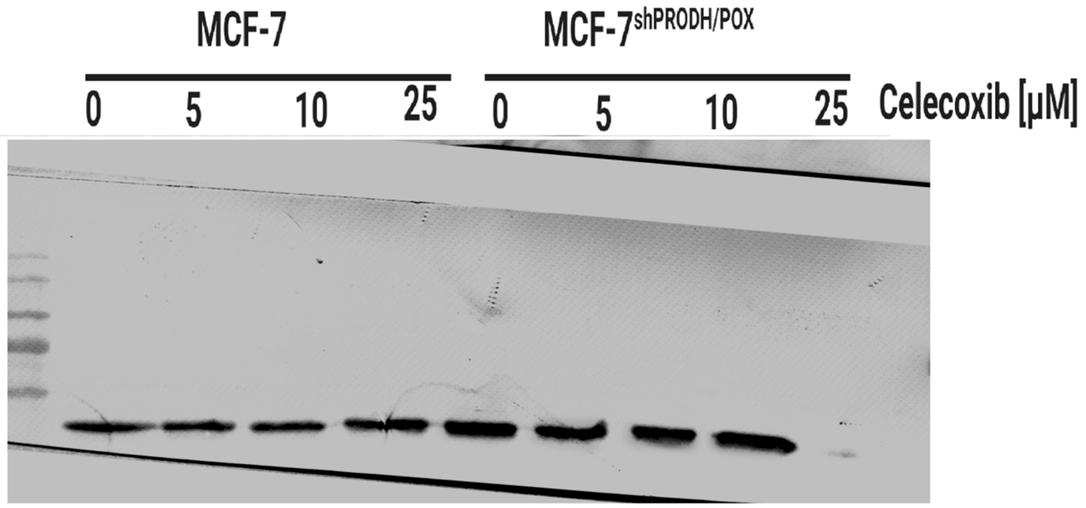

Beclin 1 protein expression

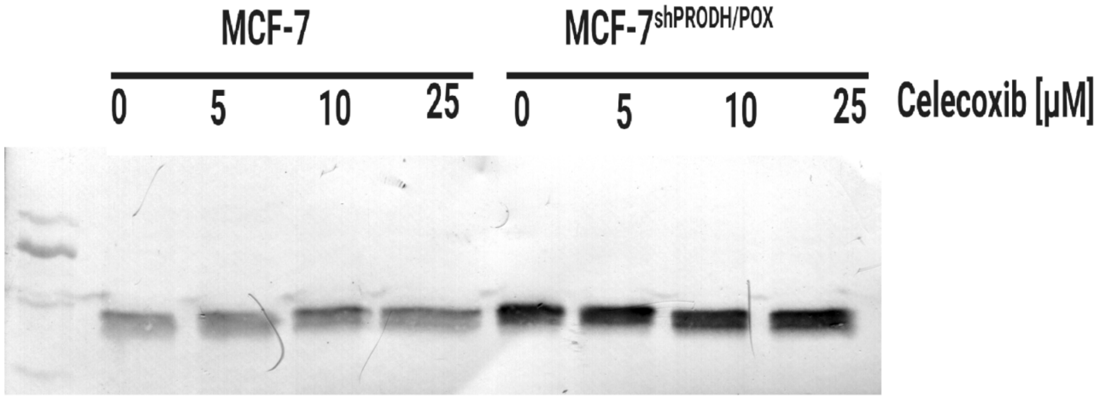

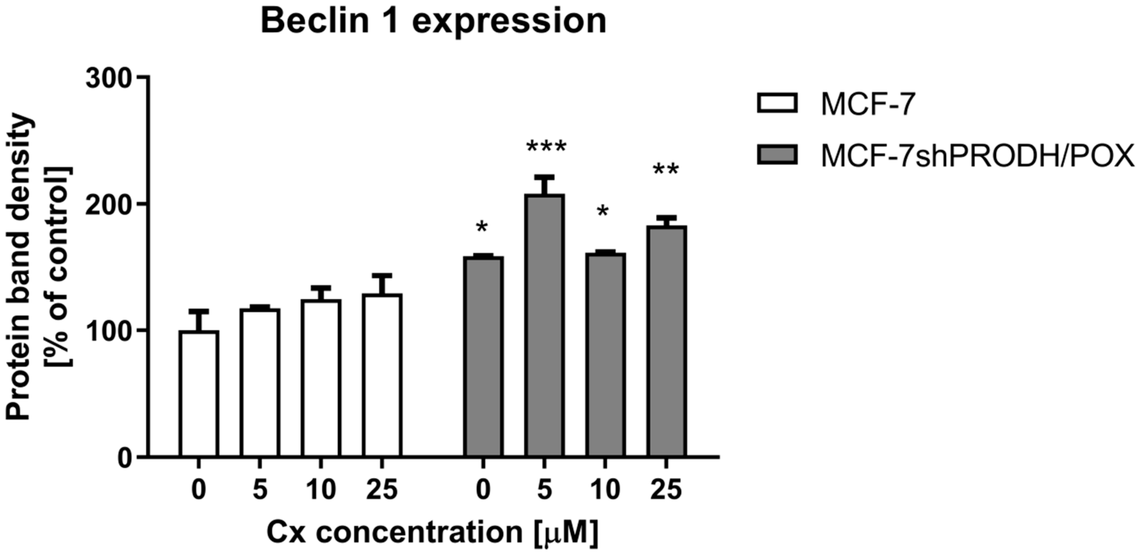

LC3A/B protein expression

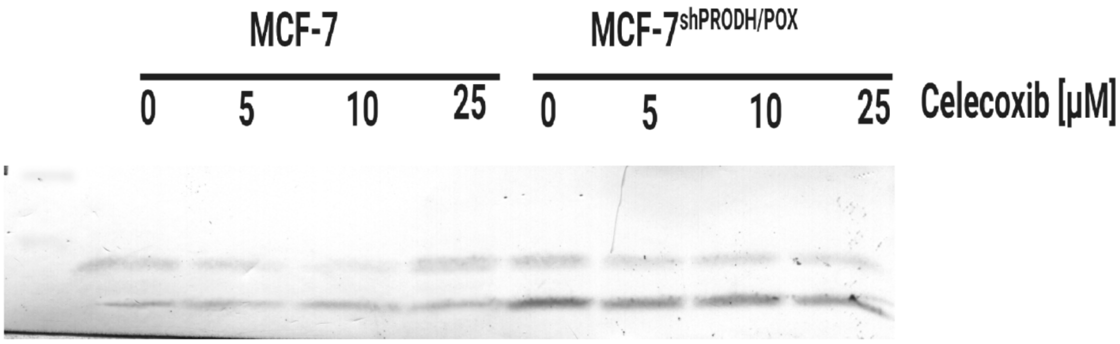

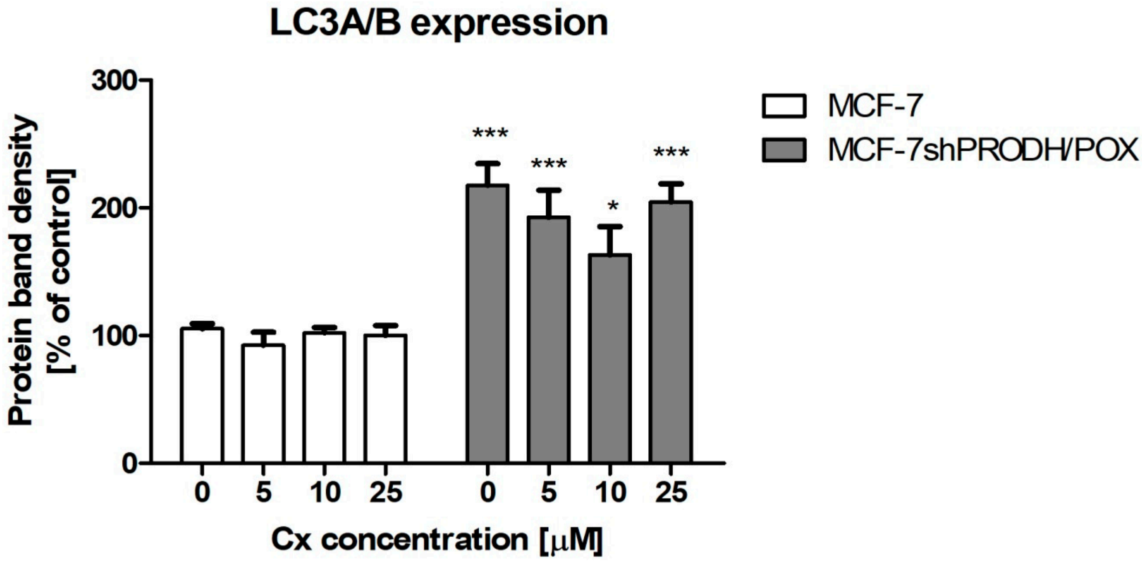

GAPDH protein expression

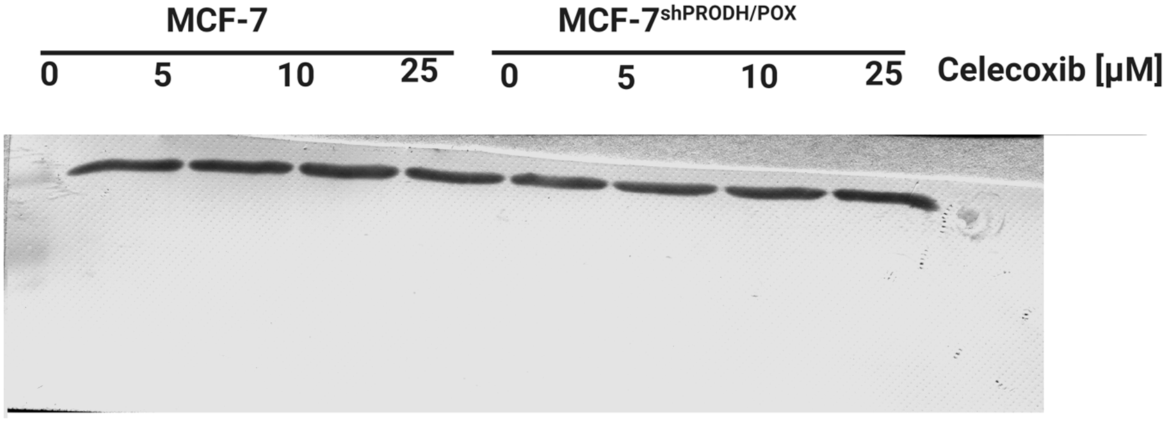

Figure S4. Western immunoblots with densitometric analysis presented in Fig. 2.

PRODH/POX protein expression

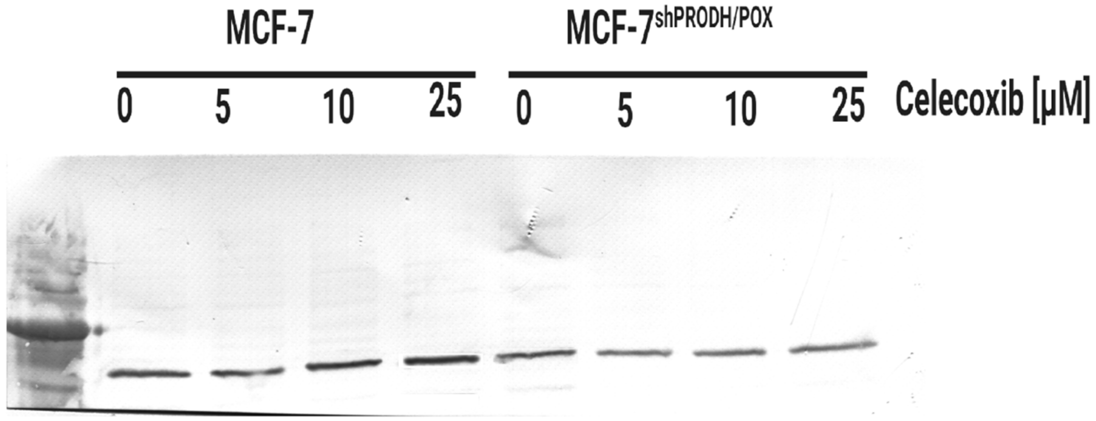

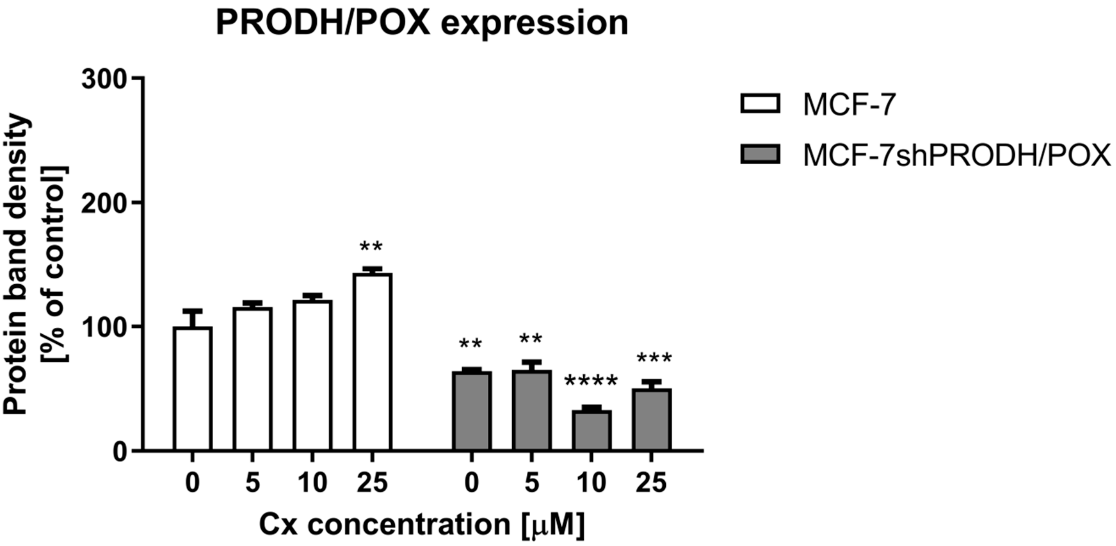

PYCR1 protein expression

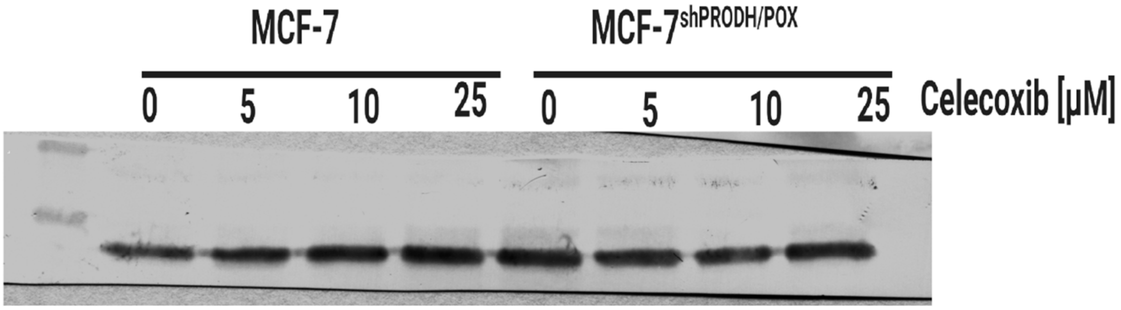

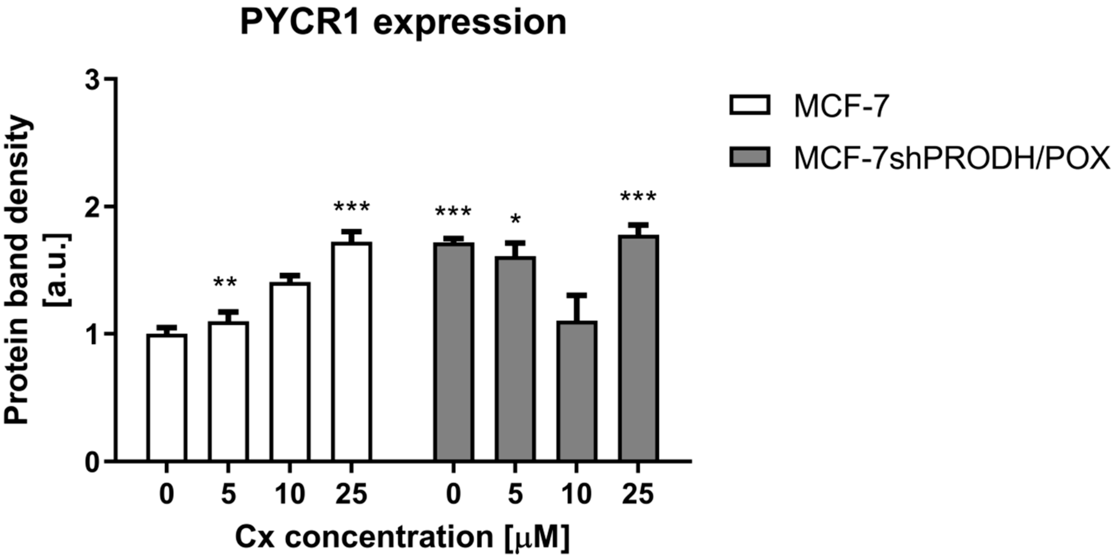

PYCR2 protein expression

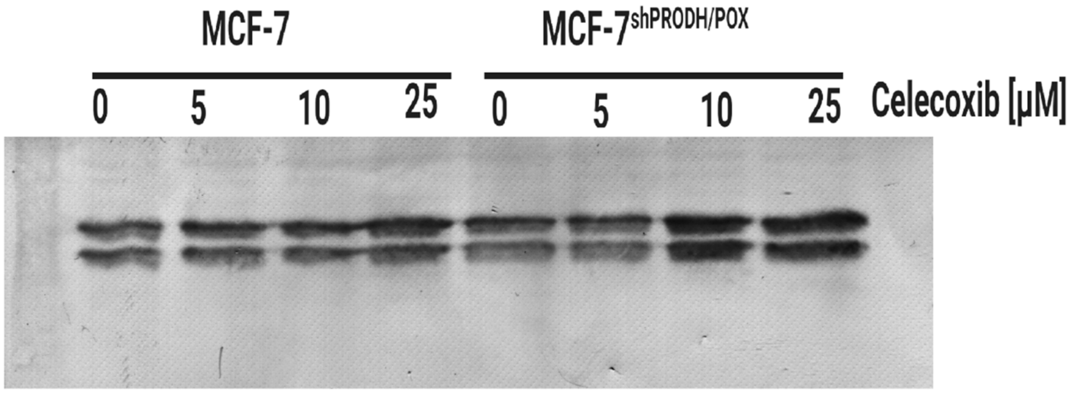

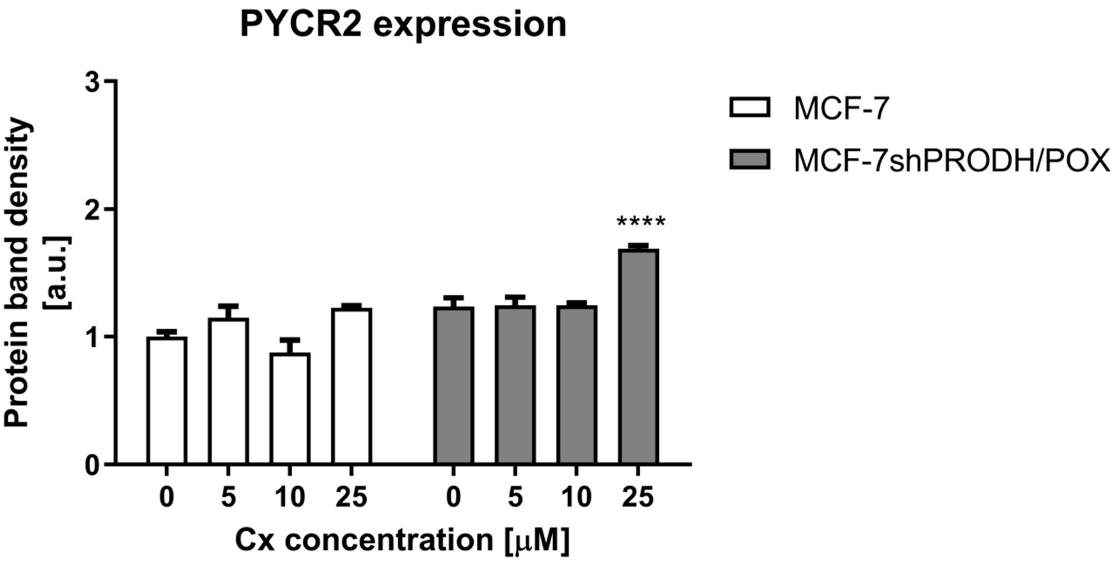

GAPDH protein expression

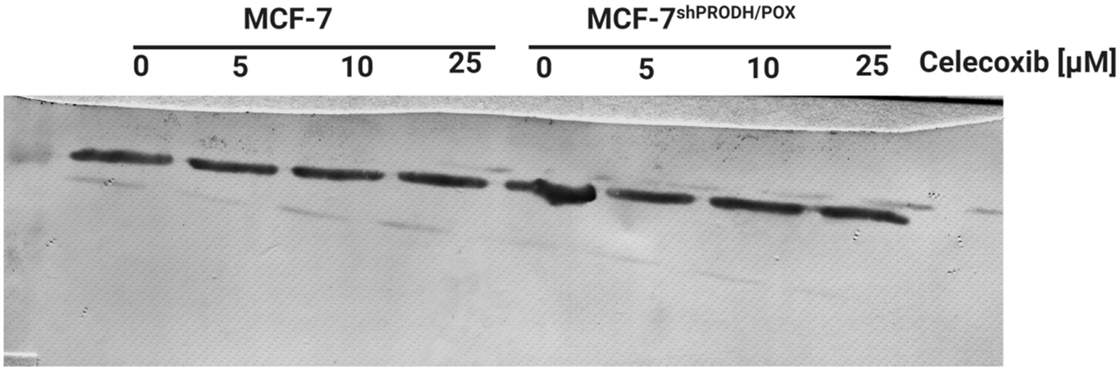

Figure S5. Representative blots with densitometric analysis presented in Fig. 3.

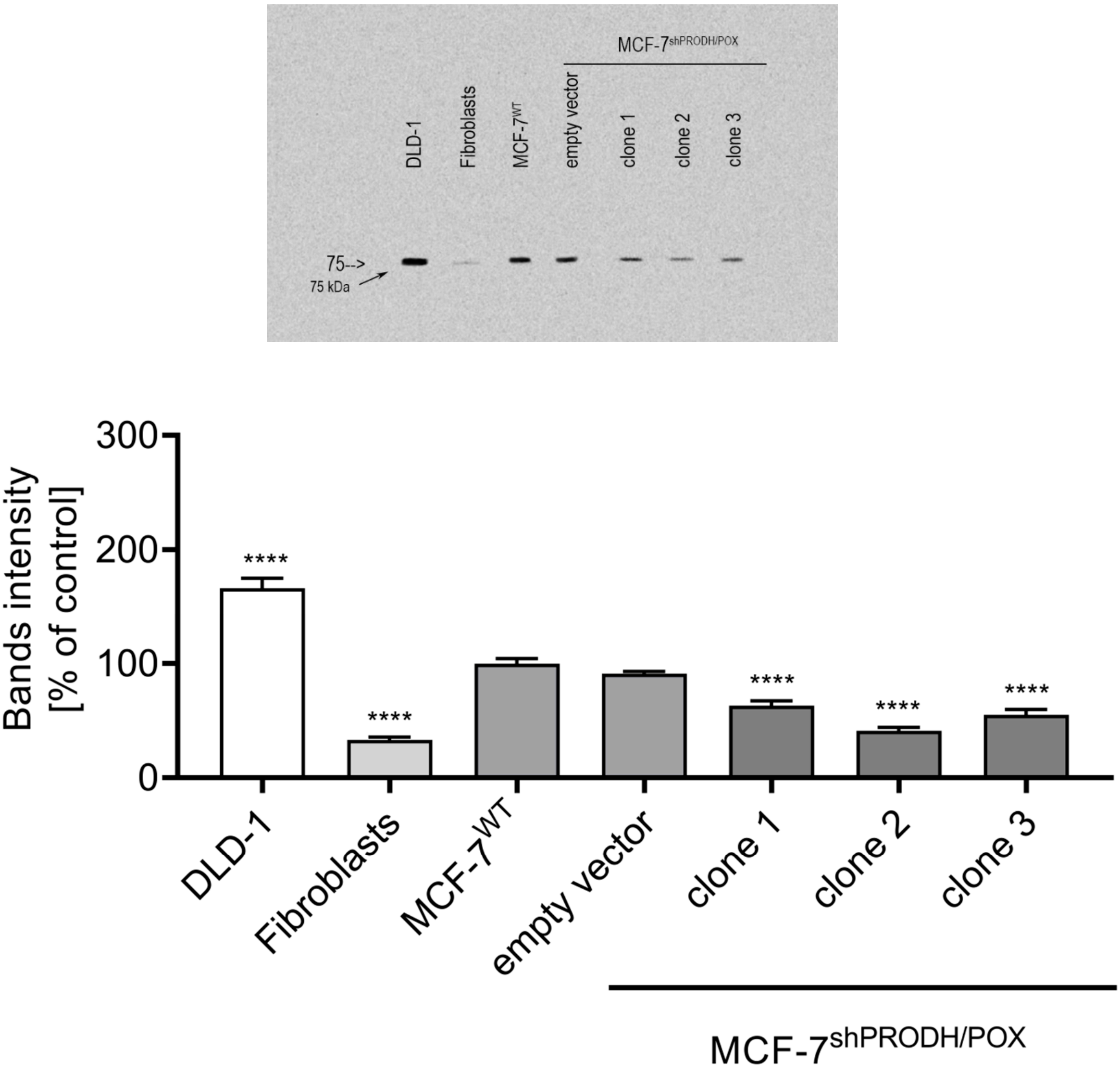

Figure S6. Efficacy of shRNA-based PRODH/POX knock-down in MCF-7 cells. PRODH/POX protein expression in non-treated DLD-1, fibroblasts, MCF-7, and MCF-7$^{shPRODH/POX}$ cell lysates analyzed by Western immunoblotting as previously published [1]. DLD-1 cells were used as a positive control and fibroblasts as a negative control for the expression of PRODH/POX. Transfection of the MCF-7cells with different PRODH/POX shRNA constructs (clone 1-3) were done Representative blot is presented and the intensity of POX bands was quantified by densitometry and normalized to GAPDH, values represent the mean (% of control) ± SD of three experiments, *P <0.001.

References

1. Zareba, I.; Surazynski, A.; Chrusciel, M.; Miltyk, W.; Doroszko, M.; Rahman, N.; Palka, J. Functional Consequences of Intracellular Proline Levels Manipulation Affecting PRODH/POX-Dependent Pro-Apoptotic Pathways in a Novel in Vitro Cell Culture Model. *Cell Physiol Biochem* **2017**, *43*, 670-684, doi:10.1159/000480653.
